# Case report: Development of central precocious puberty in a girl with late-diagnosed simple virilizing congenital adrenal hyperplasia complicated with Williams syndrome

**DOI:** 10.3389/fendo.2024.1352552

**Published:** 2024-04-18

**Authors:** Eun Young Joo, Myung Ji Yoo, Su Jin Kim, Woori Jang, Ji-Eun Lee

**Affiliations:** ^1^ Department of Pediatrics, Inha University College of Medicine, Inha University Hospital, Incheon, Republic of Korea; ^2^ Department of Laboratory Medicine, Inha University College of Medicine, Inha University Hospital, Incheon, Republic of Korea

**Keywords:** case report, congenital adrenal hyperplasia, simple virilizing type, central precocious puberty, late diagnosis, Williams syndrome

## Abstract

Congenital adrenal hyperplasia (CAH) and Williams Syndrome (WS; MIM # 194050) are distinct genetic conditions characterized by unique clinical features. 21-Hydroxylase deficiency (21-OHD; MIM #201910), the most common form of CAH, arises from mutations in the *CYP21A2* gene, resulting in virilization of the external genitalia in affected females, early puberty in males, and short stature. Williams syndrome, caused by a microdeletion of 7q11.23, presents with distinctive facial features, intellectual disability, unique personality traits, early puberty, and short stature. This case report describe the clinical features of a 4-year-old girl referred due to progressive virilization and developmental delay. Genetic analysis confirmed concurrent CAH and WS, identifying a novel mutation in the *CYP21A2* gene (c.1442T>C). Following corticosteroid therapy initiation, the patient developed central precocious puberty. This case report delves into the pubertal change patterns in a patient affected by overlapping genetic conditions, providing valuable insights in to the intricate clinical manifestation and management of these rare complex disorders.

## 1 Introduction

Congenital adrenal hyperplasia (CAH) comprises a family of autosomal recessive disorders characterized by impaired cortisol synthesis from cholesterol by the adrenal cortex, thereby affecting the production of glucocorticoids, mineralocorticoids and sex hormones. The most common type of CAH is 21-Hydroxylase deficiency (21-OHD, MIM #201910), which result from mutations in the *CYP21A2* gene. Notably, 21-OHD is classified into classic and non-classic CAH, with classic CAH further subdivided into salt-losing and simple virilizing types. Typically, patients with classic CAH exhibit adrenal insufficiency, hyperpigmentation due to increased adrenocorticotropic hormone (ACTH) levels, and virilization in the female due to excessive adrenal androgen biosynthesis (1, 2). Most patients with classic CAH are diagnosed during infancy through newborn screening (NBS) for 17-hydroxyprogesterone (17-OHP). Growth, precocious puberty, and obesity are notable concerns in pediatric patients with CAH, given the need for lifelong steroid use following diagnosis. The estimated prevalence of classic CAH is 1 in 10,000– 20,000 live births (3, 4).

Williams Syndrome (WS) (synonym: Williams-Beuren Syndrome, MIM # 194050) is a genetic developmental disorder caused by a microdeletion of 7q11.23. WS is characterized by developmental delay, distinctive facial features, unique personality characteristics, cardiovascular disease, and renal and urinary tract abnormalities (5). Short stature and early puberty are additional concerns in children with WS. The prevalence of WS is estimated to affect approximately 1 in 7,500-10,000 individuals (5, 6).

The Concomitant occurrence of multiple genetic disorders is not uncommon. Molecular analysis has revealed that 4.9% of patients are diagnosed with multiple genetic disorders (7).. In this case, we identified the simultaneous presence of CAH and WS by focusing on developmental delays, which are rare in CAH cases.

We present a rare case of concurrent CAH and WS in a patient exhibiting progressive clitoromegaly, premature pubarche, and developmental delay. This case may shed light on the pubertal characteristics of a patient with simultaneous CAH and WS.

## 2 Case presentation

A 4-year- and-2-month-old girl was referred for evaluating precocious development of pubic hair and progressive clitoromegaly over the past 6 months. She displayed rapid height growth and skin hyperpigmentation over the course of one to two years. Despite experiencing developmental delays such as incomplete toilet training and delayed language development, she exhibited a unique personality including overfriendliness and social disinhibition.

She was born to healthy and non-consanguineous parents (Korean father and Vietnamese mother) at 40 weeks of gestational age and a birth weight of 2.8 kg (16.2 percentile for gestational age). There was no history of genital ambiguity or intellectual disability in any family member, including siblings.

Based on physical examination at birth, the patient had typical female external genitalia with no signs of virilization or hyperpigmentation. The NBS result for classical CAH was within the normal range (17-OHP 2.97 ng/mL, reference ≤ 6.0 ng/mL). At the first visit, her height and weight measured 108.5 cm (Height-z 1.18) and 18.2 kg (Weight-z 0.69), respectively. The patient exhibited distinctive facial features, including acne on the forehead, bitemporal narrowing, periorbital fullness, a wide mouth, a small chin, a broad nose with microdontia, and malocclusion. While facial acnes, pubic hair growth, and hyperpigmentation were present, no breast engorgement was observed (sexual maturity rating [SMR] B1 PH2). Genital examination revealed clitoromegaly, with the clitoris measuring 25 mm in length and 15 mm in diameter (mean clitoral diameter 3.4 mm in 4-8years old (8), (Prader scale 1).

**Figure 1:**
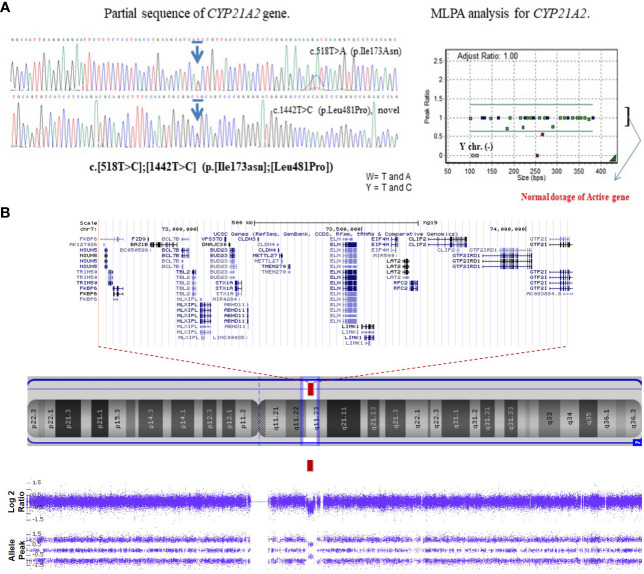
Genetic analysis of the patient. **(A)** The patient has a compound heterozygote variant, in *CYP21A2* c.518T>C, p. Ile173Asn in Exon 4 (pathogenic variant), c.1442T>C, p. Leu481Pro in exon 10 (novel likely- pathogenic variant) based on sequencing chromatogram/multiplex. Multiplex ligation dependent probe amplification (MLPA) shows two copies of the active gene. **(B)** Chromosomal microarray analysis shows a 1.4 Mb heterozygous deletion on 7q11.23 in the patient: arr[GRCh37] 7q11.23(72700524_74143060) x1. Blue arrows mean the mutation site.

A 60-minute ACTH stimulation test confirmed the diagnosis of primary adrenal insufficiency. (ACTH basal 306 pg/mL, cortisol peak 4.79 μg/dL, 17-OHP peak > 150 ng/mL) Aldosterone and plasma renin activity (PRA) were elevated without serum electrolyte imbalance (**Table 1**). The patient had a 46, XX karyotype. Molecular analysis of *CYP21A2* was conducted using multiplex ligation-dependent probe amplification (MLPA) and sequence-specific differential PCR amplification of the *CYP21A2* and *CYP21A1P* genes (9). Compound heterozygote variants were identified, including a pathogenic variant of c.518T>A (p. Ile173Asn) and a novel likely-pathogenic variant of c.1442T>C (p. Leu481Pro) in the *CYP21A2* gene. (**Figure 1A**). The classic CAH, potentially of the simple virilizing type, was diagnosed based on the results of the 60-minute ACTH stimulation test, *CYP21A2* mutation genotype, and biochemical abnormalities. Further genetic testing with the familial members was not pursed due to their refusal. The patient’s bone age was found to be advanced by over three years. Abdominal Magnetic resonance imaging (MRI) showed normal ovaries, uterus, and adrenal glands, except for malrotation of the right kidney. The patient was diagnosed with moderate intellectual disability (K-WPPSI-IV, FSIQ 45; classified as extremely low: <70). The diagnosis of WS was confirmed through chromosomal microarray analysis with the CytoScan Dx Assay (Thermo Fisher Scientific., Santa Clara, CA, USA), which identified a 1.4-MB microdeletion in 7q11.23 (**Figure 1B**).

**Table 1:** Changes in physical examination findings and laboratory parameters.

Laboratory exam		Reference range	At diagnosis (4yr 2mo)		After 6mo (4yr 8mo)	After 1 yr (5yr 2mo)
Height-z (cm)			1.18 (108.5)		1.38 (113.3)	1.54 (116.2)
Weight-z (kg)			0.69 (18.2)		1.09 (20.7)	1.8 (24.0)
BMI-z (kg/m2)			- 0.22 (15.5)		0.30 (16.1)	1.34 (17.8)
SMR			B1 PH2		B2 PH2	B3 PH2
Clitoromegaly (length x diameter)			25 mm x 15 mm		25 mm x 10 mm	20 mm x 4 mm
△BA-CA (years)			3.75		4.25	4.75
Electrolyte	Na	133–145 (mEq/L)	137.7		135.6	137.0
	K	3.5–5.5 (mEq/L)	4.47		4.16	4.19
ACTH (8am)		10–60 (pg/mL)	306.2		33.5	44.70
17-OHP		0.03–0.9 (ng/mL)	50.7		16.63	31.8
Aldosterone		3–35 (ng/dL)	45.64		19.7	41.63
Plasma renin activity		0.5–5.8 (ng/mL/hr)	7.76		5.29	5.02
ACTH stimulation test	Cortisol (0/30/60min)	Peak cortisol ≥ 18 μg/dL	0 min 30 min 60 min	5.18 5.48 4.79	–	–
	17-OHP (ng/mL)		0 min 30 min 60 min	118.9 >150 >150		
DHEAS		5–57 (μg/dL)	99.7		40.3	32.5
Testosterone		0.03–0.1 (ng/mL)	0.49		0.32	0.28
Estradiol		< 15 (pg/mL)	11.78		35.62	34.0
GnRH stimulation test	LH (mIU/mL)	basal/peak	2.0/4.23		1.21/3.83	1.04/5.29
	FSH (mIU/mL)	basal/peak	3.02/14.97		4.87/13.83	4.11/11.07
Treatment	Hydrocortisone	(mg/m2/day)	13		18	18
	Fludrocortisone	(mg)			0.05	0.1
	GnRH agonist					Start

SMR, sexual maturity rating; BA, bone age; CA, chronological age; ACTH, adrenocorticotrophic hormone; LH, luteinizing hormone; FSH, follicle-stimulating hormone; 17-OHP, 17- hydroxyprogesterone; DHEAS, dehydroepiandrosterone sulfate; GnRH, gonadotropin releasing hormone; Na, sodium; K, potassium.

Two months after starting hydrocortisone therapy (13 mg/m2/day), the intensity of hyperpigmentation decreased gradually, and acne occurrence became less frequent. To ensure the effective suppression of adrenal androgens, the hydrocortisone replacement dosage was appropriately adjusted (up to 18 mg/m2/day) over the course of six months. Fludrocortisone was introduced to reduce the administered hydrocortisone dose and achieve biochemical control of elevated PRA levels. During six months’ treatment period, the patient experienced breast engorgement (SMR B2 B2), which coincided with the cessation of progressive pubarche and a reduction in clitoromegaly (from 15 mm to 10 mm in diameter). After one year of hydrocortisone therapy, the patient exhibited an accelerated growth rate, rapid weight gain, further advanced bone age, and progressive precocious thelarche. A gonadotropin-releasing hormone (GnRH) stimulation test was performed, revealing results consistent with central precocious puberty (**Table 1**). Subsequently, GnRH analogue(GnRHa) therapy was initiated.

## 3 Discussion

In this study, we present a case of concurrent 21-OHD CAH and WS, aiming to evaluate pubertal changes in the affected patient. The gene implicated in 21-OHD CAH, *CYP21A2*, is located on chromosome 6p21.3, whereas the locus for WS is located on chromosome 7q11.23. Both genetic disorders are associated with early puberty and short stature. However, developmental delays and renal anomalies are not typical feature of CAH patients. Given the presence of multiple atypical clinical features in our patient, we investigated the possibility of other concomitant genetic disorders. Advance in accurate diagnosis may lead to improved prognosis through the implementation of appropriate management strategies.

CAH is the most common cause of atypical genitalia among female patients with the normal 46, XX karyotype. In the present case, the early diagnosis was delayed until virilization in external genitalia was observed, as the patient initially had normal NBS results and typical female external genitalia at birth. The false-negative rate of NBS, using 17-OHP for classical CAH, ranges between 2% and 9%. This rate tends to be higher in patient with the simple virilizing form (10). In the case, compound heterozygote with variants in the *CYP21A2* gene was confirmed. While one variant, c.518T>A (p.Ile173Asn), is a known pathogenic variant associated with the simple virilizing type, the other mutation, c.1442T>C (p.Leu481Pro), is a novel likely-pathogenic variant. Based on the patient’s endocrinological characteristics, the p.L481P variant was considered indicative of the simple virilizing type of classic CAH. However, further research is required to comprehensively elucidate its implications.

Based on patient’s intellectual disability, we considered the possibility of other coexisting disorders, leading to a diagnosis of WS. Individuals with CAH typically have normal neurodevelopment and intelligence, except in cases of salt-wasting adrenal crisis-induced brain damage (11, 12). However, children diagnosed with WS demonstrated a wide range of intellectual disabilities, range from profound impairment to nearly normal functioning (5). In the current case report, the patient also presented with delayed language development. Typically, individuals with WS exhibit better verbal ability compared to nonverbal abilities although this difference tends to be less pronounced in children compared to adults (13)

Early or precocious puberty can present as a manifestation of both CAH and WS. CAH is a prevalent cause contributing to peripheral precocious puberty. It is well-established that excessive androgens levels precipitate early puberty and epiphyseal fusion (2). In patient with CAH, delayed diagnosis and inadequate control of adrenal androgen secretion are risk factors for the progression to central precocious puberty. Moreover, chronic hyperandrogenemia has the potential to activate the hypothalamic-pituitary axis. However, untreated female CAH patients may not initially exhibit early breast budding due to the suppressive effects of hyperandrogenemia on the development of breast tissue. In a previous case study, signs of breast development were reportedly observed following continued treatment (14). The authors observed a delayed diagnosis, with initial absence of breast engorgement, findings similar to those of the current case report. However, they also noted progression to central precocious puberty following treatment. Proper glucocorticoid treatment has been reported to induce a sudden decrease in androgen levels, thereby stimulating the pituitary gland to release gonadotropins (1) Therefore, it is crucial to recognize that initiating corticosteroid treatment may trigger central precocious puberty during the management of patients with late- diagnosed CAH.

Early puberty has been reported in up to 50% of patient with WS, whereas central precocious puberty has been noted in 3-18% of these individuals (5, 15, 16).. The mechanism underlying pubertal changes in WS is yet to be fully elucidated. Approximately 50% of patients with WS present with short stature, which is evident and becomes more apparent over time. Patients with WS tend to have a low birth weight, experience decreased linear growth in childhood and early puberty, and undergo a reduced growth spurt in adolescence (15, 17)

In both CAH and WS, pubertal changes may contribute to short stature, and GnRHa therapy has been shown to improve final adult height in females with secondary central precocious puberty (15, 18, 19). Patients with CAH may initially appear taller than those without CAH at the onset of puberty; however, a meta-analysis has indicated that the final adult height tends to be shorter (Height-z: -1.38 SDS) (20–22). Growth impairment in patients with CAH may be attributed to glucocorticoid therapy and precocious puberty. Early diagnosis of CAH can enhance the prognosis of precocious puberty and the patient’s final adult height. Irrespective of the specific type of CAH, mineralocorticoids may offer potential benefits in terms of enhancing final adult height by reducing the overall dosage of glucocorticoid (22). In the present case, mineralocorticoids therapy was utilized to promote appropriate growth and mitigate excessive weight gain by reducing the dosage of glucocorticoid.

## 4 Conclusion

To the best of our knowledge, this is the first case report detailing late-diagnosed simple virilizing CAH alongside concomitant WS. To mitigate underdiagnoses and enhance prognostic outcomes, it is imperative to contemplate concomitant disorders presenting with atypical multiple clinical features.

## Data availability statement

The datasets for this article are not publicly available due to concerns regarding participant/patient anonymity. Requests to access the datasets should be directed to the corresponding author.

## Ethics statement

The studies involving humans were approved by the Inha University Hospital IRB (No.2023-09-008). The studies were conducted in accordance with the local legislation and institutional requirements. Written informed consent for participation in this study was provided by the participants’ legal guardians/next of kin. Written informed consent was obtained from the minor(s)’ legal guardian/next of kin for the publication of any potentially identifiable images or data included in this article.

## Author contributions

EJ: Conceptualization, Data curation, Methodology, Writing – original draft, Writing – review & editing. MY: Data curation, Writing – review & editing. SK: Conceptualization, Data curation, Methodology, Writing – review & editing. WJ: Data curation, Formal analysis, Writing – review & editing. J-EL: Conceptualization, Data curation, Writing – review & editing.
